# Fluorescence spectroscopy as a novel tool in hematological diagnostics

**DOI:** 10.1063/5.0264155

**Published:** 2025-04-01

**Authors:** Nugzar Gomidze, Lali Kalandadze, Miranda Khajishvili, Omar Nakashidze, Izolda Jabnidze, Davit Jakobia, Kakha Makharadze

**Affiliations:** Batumi Shota Rustaveli State University, Batumi, Georgia

## Abstract

The present paper explores the application of fluorescence spectroscopy in erythrocyte analysis, aiming to enhance spectral diagnostics in biomedical research. The primary objective is to develop innovative methodologies for improving the precision of hematological diagnostics and disease monitoring. Utilizing 3D fluorescence spectroscopy and excitation/emission wavelength mapping, erythrocyte samples are examined across multiple wavelengths, generating distinct spectral profiles that reveal biochemical composition, oxygenation status, and metabolic alterations. Advanced data analysis enables the identification of pathological changes in erythrocytes, contributing to a more comprehensive diagnostic approach. Additionally, this study integrates fluorescence spectroscopy with traditional clinical hematological analysis, comparing spectroscopic findings with complete blood count parameters for two patients. Blood samples were subjected to fluorescence analysis under deuterium, halogen, and ultraviolet excitation sources, allowing for a detailed correlation between spectroscopic biomarkers (hemoglobin, deoxyhemoglobin, and plasma characteristics) and clinical parameters (hemoglobin concentration, hematocrit, and red blood cell indices). The findings demonstrate that fluorescence spectroscopy provides complementary diagnostic insights, detecting subtle physiological variations in blood composition that conventional methods might overlook. By integrating these two diagnostic approaches, this research highlights the potential of fluorescence-based techniques as a noninvasive and efficient tool for hematological diagnostics.

## I. INTRODUCTION

Fluorescence spectroscopy is one of the most important techniques used across various fields, including blood analysis, biological sample examination, and other chemical and physical studies. This method is particularly notable for its ability to perform precise analysis with minimal invasiveness. In forensic science, fluorescence spectroscopy is essential for analyzing bloodstains, enhancing blood visibility, and determining its age, which is crucial for forensic investigations.^1^

The fundamental principles of this technique have been described in detail, illustrating how fluorescence spectroscopy operates and how it is applied in pharmaceutical sciences.^2^ Its application is not limited to biological samples alone; fluorescence spectroscopy is also widely used in bioanalysis, where it aids in achieving high sensitivity and specificity.^3^

Fluorescence-activated cell sorting (FACS) and methods for studying red blood cells are also extensively applied in molecular biology and medical research. These methods allow for the separation and analysis of cells based on fluorescence characteristics, significantly contributing to cellular research that requires identifying and analyzing various cell characteristics and functions.^4^

Research into the binding of quinacrine to DNA (deoxyribonucleic acid) using fluorescence demonstrates that fluorescence methods are indispensable for studying drug–DNA interactions, a crucial area in pharmacology and cancer research.^5^

Additionally, the influence of zeta potential on the microrheological properties of erythrocytes provides essential information for analyzing cell fluorescence in liquid media.^6^ Furthermore, the development of a new algorithm designed for analyzing fluorescence data in wine highlights fluorescence's broad applications in the study of biological and chemical samples.^7^

Thus, the level of development in fluorescence spectroscopy research reflects its interdisciplinary nature and its critical role in modern scientific investigations.

The StellarNet BlackComet spectrometer is an instrument ideally suited for examining fluorescence spectra in the UV-Vis range. It can be applied to several research topics mentioned above. The BlackComet is capable of analyzing fluorescence spectra, which is valuable for studying various biological, chemical, and physical samples.

To study the microrheological properties of erythrocytes, the StellarNet BlackComet can closely examine the fluorescent characteristics of cells in a liquid medium. Additionally, this spectrometer can be used to investigate the influence of zeta potential, which is important in biological and physiological processes.

Tang *et al.* explored near-infrared (NIR) fluorescence in erythrocyte-mimicking particles, focusing on fluorescence stability, quantum yield, and integrated fluorescence emission.^8^ These properties provide insights into the intensity and distribution of fluorescence emission, helping to simulate erythrocyte properties in studies requiring high stability and specific fluorescence characteristics and physical properties using absorption imaging.

Paul *et al.* used quantitative absorption imaging to assess the physical and mechanical properties of single erythrocytes. This method, sensitive to changes in erythrocyte mechanical properties, allows differentiation between cells based on age, flexibility, and other structural characteristics, which is valuable in diagnostics for conditions like anemia or sickle cell disease.^9^

Ramanujam's work emphasizes the use of fluorescence spectroscopy to quantify hemoglobin and other blood components *in vivo*.^10^ By monitoring the fluorescence intensity and emission spectra, researchers can assess oxygen transport efficiency and the health status of red blood cells. This can be applied to detect conditions affecting oxygen delivery and hemoglobin functionality.

Lee and Yeung analyzed individual erythrocytes using laser-induced fluorescence detection.^11^ They noted significant differences in fluorescence intensity across erythrocytes, correlating with cell age and possible protein variations. This approach allows for precise single-cell analysis, offering insight into erythrocyte life cycle stages and the distribution of cellular health within populations.

Saldanha's study reviewed instrumental methods, including light scattering and fluorescence, to measure erythrocyte shape, size, and internal composition.^12^ This approach provides data on erythrocyte deformability, a key factor in understanding circulatory health and assessing diseases that impact erythrocyte morphology.

Current diagnostic methods, such as microscopic analysis and traditional biochemical tests, play an important role in assessing the health and functional state of erythrocytes. However, they have certain limitations: **traditional methods often require extensive processing, advanced medical expertise, and specialized equipment, which can make the diagnostic process less efficient**.

In contrast, fluorescence spectroscopy offers a noninvasive, rapid, and highly effective means of analysis. This method provides unique fluorescence profiles of cells and biomarkers based on excitation and emission wavelengths, enabling differentiated diagnostics. By employing comprehensive 3D fluorescence spectroscopy (3DF) and excitation/emission wavelength analysis (AEM), precise markers of diseases can be identified, ensuring more accurate and faster diagnostics compared to traditional methods.

The aim of this study is to enhance the application of fluorescence spectroscopy in erythrocyte diagnostics by introducing new methodologies that allow for the detection of pathological changes in the biochemical and structural characteristics of cells.

## II. SPECTROMETER SCHEMATIC AND METHODOLOGY

The schematic of the StellarNet BlackComet spectrometer consists of several key components that enable it to collect and analyze light spectra. The following are the main components and their functions:

The light source generates light directed at the sample under analysis. This light can be from the ultraviolet (UV), visible (Vis), or near-infrared (NIR) spectrum, depending on the application. For example, UV light might be used in biological sample analysis to induce fluorescence in the sample.

The sample is placed in a specialized holder that ensures proper transmission of light through it. The light interacting with the sample is either reflected or transmitted, allowing the spectrometer to capture its fluorescence or reflected spectrum.

The optical fiber connects the light source and the sample to the spectrometer. It transmits or reflects light from the sample to the spectrometer. The fiber can vary in length and diameter, depending on the specific research needs.

The monochromator uses a grating system to separate light into different wavelengths. This process disperses the light received from the sample into a spectrum of colors based on wavelength.

The detector records the intensity of light at various wavelengths. The BlackComet spectrometer uses a CCD (charge-coupled device) detector, which is highly sensitive and allows precise signal detection across different wavelengths. The CCD detector captures the intensity data, which is then used for analysis.

**FIG. 1.**
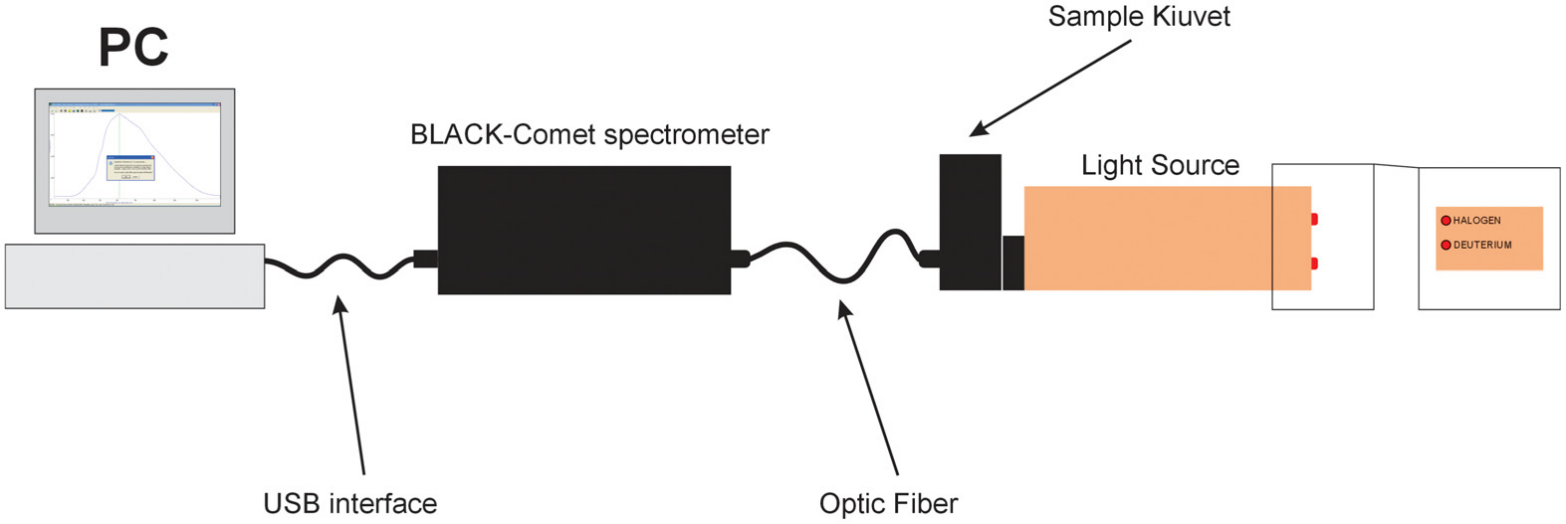
The principal scheme of the spectrometric system.

The data collected by the detector are sent to a computer or data processing system for spectral analysis. Here, it is possible to perform a detailed examination of the sample's fluorescence properties and composition (Fig. 1).

The BlackComet spectrometer collects the spectrum of light emitted from the sample and passes it through the monochromator, which separates the light into different wavelengths. The CCD detector records the intensity associated with each wavelength and transmits these data to a computer, where it is visually displayed and analyzed.

This setup allows the spectrometer to detect and analyze various samples' fluorescence and reflected light, making it valuable for research across biological, chemical, and materials science fields.

To enhance the accuracy and reliability of this study, standard calibration procedures were used for the **StellarNet BlackComet spectrometer**. The spectrometer was calibrated using known emission lines, ensuring that the equipment accurately responded to the required excitation and emission wavelengths.

To assess reproducibility and data accuracy, control samples were selected and their spectral characteristics were systematically verified. These control samples, consisting of standard compounds, aided in evaluating the validity of the sample measurements, allowing for consistent data comparison.

## III. QUANTITATIVE CHARACTERISTICS FOR ERYTHROCYTE ANALYSIS

The mathematical foundation of fluorescence spectroscopy theory is primarily based on analyzing the relationship between light intensity and wavelength, which helps determine the fluorescent characteristics of a sample. Here are some key mathematical aspects of this theory:

The **fluorescence intensity** I depends on the sample **concentration** C, the **excitation light intensity** $I_{0}$, and the excitation wavelength. This relationship is often represented by the following formula:

$$I=\Phi \cdot I_{0}\cdot C\cdot \epsilon ,$$ (1)

where Φ is the fluorescence quantum yield, indicating the fraction of absorbed photons that are re-emitted as fluorescence, and ϵ is the **absorption coefficient**, which varies with wavelength.

The shape of the fluorescence spectrum usually depends on the **absorption** and **excitation wavelengths**. The absorption coefficient ϵ(λ) is closely related to the **absorption spectrum**, which is defined by the sample's **absorbance** at specific wavelengths,

$$A(\lambda )={\text{log}}_{10}\left(\frac{I_{0}}{I}\right)=\epsilon (\lambda )\cdot C\cdot l,$$ (2)

where A(λ)—the absorbance at wavelength λ and l—the path length of light through the sample.

**Stokes shift** is the difference between the peak wavelengths of absorption and fluorescence emission. Measuring this shift provides important information about the environment of the fluorescent molecule and its quantum efficiency. It is calculated as

$$\Delta \lambda =\lambda_{\text{emission}}-\lambda_{\text{absorption}},$$ (3)

where $\lambda_{\text{emission}}$ and $\lambda_{\text{absorption}}$ represent the peak wavelengths of fluorescence emission and absorption, respectively.

The **total integrated fluorescence intensity** is sometimes calculated across all wavelengths in the emission spectrum,

$$I_{\text{total}}=\int_{\lambda_{\text{min}}}^{\lambda_{\text{max}}}I(\lambda )\;d\lambda .$$ (4)

This calculation provides the sample's overall fluorescence signal, which can be used to determine concentration or other properties.

Some applications of fluorescence spectroscopy require calculating the **penetration depth (** d**)** of light in sample layers, which depends on the wavelength and the optical properties of the sample. It can be calculated as

$$d=\frac{1}{\epsilon \cdot C}.$$ (5)

The **shape factor** provides insight into the morphology of erythrocytes, particularly whether they maintain a typical biconcave disk shape or have deviated due to pathological conditions. This factor can be derived from light scattering data, often focusing on the intensity and distribution of scattered light,

$$\mathrm{Shape}\;\mathrm{Factor}=\frac{I_{\text{peak}}}{R},$$ (6)

where $I_{\text{peak}}$—the peak intensity of the scattering signal and R—the average diameter of the erythrocyte.

A lower shape factor suggests a more biconcave shape, while higher values may indicate spherical or distorted cells.

The **deformability index (DI)** reflects the erythrocyte's ability to deform as it passes through narrow capillaries, which is critical for circulatory health. Deformability is often measured by analyzing the spread and peak of the scattering intensity,

$$DI=\frac{\int I_{\text{scattering}}(\lambda )d\lambda }{R},$$ (7)

where $I_{\text{scattering}}(\lambda )$ represents the scattering intensity as a function of wavelength λ.

A higher deformability index indicates increased cell flexibility, while lower values suggest rigidity, which can be a marker for certain diseases like sickle cell anemia.

Generally, deformability is affected by the **zeta potential (** ζ**)** because the electrostatic repulsion between cells influences their flexibility and interaction in suspension. A reasonable assumption for the relationship between DI and ζ is a **logarithmic** or **exponential decay model,**

DI=a⋅ln(−ζ)+c, (8)

$$DI=a\cdot e^{b\zeta }+c,$$ (9)

where a and b are constants that adjust the sensitivity of DI to changes in ζ and c is a baseline deformability constant when ζ is zero or very low.

We can analyze how the surface electrostatic potential (zeta potential) affects the forces between erythrocytes. These forces, in turn, influence the tendency of erythrocytes to aggregate. The **total interaction energy** $E_{\text{total}}$ is the sum of the electrostatic repulsion and **van der Waals** attraction (between erythrocytes at a distance d),

$$E_{\text{total}}=E_{\text{rep}}+E_{\mathrm{veW}}=\pi \varepsilon R\zeta^{2}e^{-\kappa d}-\frac{AR}{12d},$$ (10)

where ε—the permittivity of the medium, R—the radius of the erythrocytes, κ—the inverse Debye length, indicating the thickness of the electric double layer, d—the distance between the surfaces of two erythrocytes, and A—the Hamaker constant for erythrocytes in a specific medium (related to the strength of van der Waals forces).

Equation (10) derived from **DLVO (Derjaguin, Landau, Verwey, Overbeek) theory** describes the overall interaction energy between erythrocytes in terms of their tendency to aggregate. When $E_{\text{total}}>0$, repulsive forces dominate, preventing aggregation. When $E_{\text{total}}<0$, attractive forces dominate, promoting aggregation.

The **size or diameter of erythrocytes** can be measured directly under a microscope or inferred from light scattering characteristics. When using light scattering, the wavelength at which scattering peaks can indicate cell size. For example, if $\lambda_{\text{peak}}$ represents the wavelength at which scattering intensity is highest, empirical relationships can be used to estimate **cell diameter**. Generally,

$$R\approx k\cdot \lambda_{\text{peak}},$$ (11)

where k is an empirically derived constant that depends on calibration.

The **internal composition index** evaluates the biochemical makeup of erythrocytes by analyzing fluorescence, specifically looking at peaks associated with cellular components like **hemoglobin** or **NADH** (**NADH—nicotinamide adenine dinucleotide hydrogen)**. It is a coenzyme found in all living cells and plays a crucial role in cellular energy production. NADH is the reduced form of **NAD^+^ (nicotinamide adenine dinucleotide)** and is involved in metabolic processes, particularly in the electron transport chain during cellular respiration, where it contributes to the generation of **ATP (adenosine triphosphate)**, the cell's main energy currency (NADH is also significant in fluorescence spectroscopy as it exhibits natural fluorescence, making it a useful marker in studying cellular metabolism and assessing cell health and function in diagnostic applications). So,

$$\mathrm{Internal}\;\mathrm{Composition}\;\mathrm{Index}=\int I_{\text{fluorescence}}\left(\lambda \right)\;d\lambda ,$$ (12)

where $I_{\text{fluorescence}}(\lambda )$ is the fluorescence intensity as a function of wavelength λ.

A higher internal composition index suggests a higher concentration of fluorescent components within the cell, providing information on the cell's metabolic state and health.

3D fluorescence analysis of erythrocytes involves creating a complete, three-dimensional profile of their fluorescent characteristics, which includes the dependency of their fluorescent signal on wavelength, fluorescence intensity, and sample volume. 3D fluorescence spectra provide detailed information about the microrheological properties of erythrocytes, which is crucial for assessing their functional state.

Let us provide a detailed description of the mathematical, quantitative, and laboratory analysis involved in the creation of 3D fluorescence spectrum.

To create a 3D fluorescence spectrum, we use the dependency of fluorescence intensity ( I) on various wavelengths and spatial dimensions (x, y, z). This dependency can be expressed by the following formula:

$$I\left(x,y,z,\lambda_{\text{emission}},\lambda_{\text{excitation}}\right)=\Phi \cdot I_{0}\cdot \epsilon \left(\lambda_{\text{excitation}}\right)\cdot C\left(x,y,z\right),$$ (13)

where $\epsilon (\lambda_{\text{excitation}})$ is the absorption coefficient.

C(x,y,z) represents the erythrocyte concentration in space ( x,y,z). The fluorescence intensity depends on the position within the sample. By mapping intensity at different points ( x,y,z), a 3D spatial profile is created, showing how fluorescence varies throughout the sample.

By measuring the total hemoglobin concentration with a spectrometer, we can estimate **RBC count** (**red blood cell count**) if we also know the **mean corpuscular hemoglobin (MCH)**—the average hemoglobin per RBC. It is a laboratory test that measures the number of red blood cells (erythrocytes) in a given volume of blood, typically reported as cells per microliter (*μ*L) or milliliter (mL). The RBC count is an important indicator of overall health, as red blood cells are responsible for transporting oxygen from the lungs to tissues throughout the body,

$$\mathrm{RBC}\;\mathrm{Count}=\frac{\mathrm{Total}\;\mathrm{Hemoglobin}\;\mathrm{Concentration}}{\mathrm{MCH}}.$$ (14)

We can use a spectrometer to measure the total hemoglobin concentration in the blood. The known MCH value is used (either from literature or a separate measurement). The RBC count is calculated using the above-mentioned formula.

MCH, which refers to the average hemoglobin content of one erythrocyte, is usually equal to 27–33 picograms (pg) in healthy people.

## IV. SPECTRAL PROPERTIES OF ERYTHROCYTES

A simple comparison of the properties of erythrocytes in different conditions, age groups, and health statuses is facilitated by the tables below. They allow us to conduct an in-depth investigation of how specific biomarkers and indices vary in different physiological and pathological conditions.

Table I presents excitation and emission wavelengths for critical erythrocyte biomarkers, including bilirubin, hemoglobin, and NADH. The data aid in identifying optimal wavelengths for fluorescence spectroscopy in erythrocyte analysis. Table II outlines the average size, shape factor, and deformability index of erythrocytes. These parameters are essential for assessing the morphology and flexibility of erythrocytes, which are relevant to circulatory health. Table III shows fluorescence intensity and spectral characteristics of erythrocytes across different age groups and health conditions. This allows for a comparative analysis of erythrocyte properties related to aging and disease.

**TABLE I.** Excitation and emission wavelengths of key fluorescent biomarkers in erythrocytes.

Excitation wavelength (nm)	Emission wavelength (nm)	Fluorescence intensity characteristics
420–500	530–570	Emission of bilirubin, varying intensities with peaks at specific intervals^13^
700	740–780	Hemoglobin mapping, moderate intensity at 700 nm excitation^14^
Near-infrared (NIR)	760–800	High fluorescence intensity, erythrocyte-mimicking particles^15^
488	520	Observed during prostaglandin E2 stimulation; moderate fluorescence intensity recorded^16^
800	820	Varied emission for RBC suspension, shifted spectra based on RBC conditions^17^

**TABLE II.** Characteristics of erythrocyte size, shape, and elasticity.

Characteristic	Description	Purpose	Typical measurement technique
Size (diameter)	Average diameter of erythrocytes (typically 6–8 *μ*m).	Assesses cell size, important for detecting anemia or other size-related abnormalities.	Microscopy and flow cytometry.
Shape factor	Ratio related to cell shape (e.g., spherical vs. biconcave).	Determines morphology, with deviations indicating potential disease (e.g., spherocytosis).	Light scattering and imaging analysis.
Deformability index	Measure of cell flexibility and elasticity.	Indicates cell's ability to deform in capillaries, vital for circulatory health.	Ektacytometry, optical tweezers, and micropipette aspiration.
Membrane rigidity	Relative stiffness of the cell membrane.	Increased rigidity can signal diseases like malaria or hereditary spherocytosis.	Micropipette aspiration and atomic force microscopy (AFM).
Volume (mean corpuscular volume—MCV)	Average cell volume (often measured in femtoliters, fL).	Helps in classifying anemia types (e.g., microcytic and macrocytic).	Automated cell counters and hematology analyzers.
Hemoglobin content (mean corpuscular hemoglobin—MCH)	Average amount of hemoglobin per erythrocyte.	Used to assess oxygen-carrying capacity.	Spectroscopy and hematology analyzers.
Surface area	Total membrane surface area, typically around 140 *μ*m^2^ per cell.	Important for gas exchange efficiency, influences oxygen delivery.	Calculated from size and shape and microscopy.
Osmotic fragility	Susceptibility of cells to hemolysis in hypotonic solutions.	Assesses membrane stability, indicating susceptibility to rupture.	Osmotic fragility test and flow cytometry.
Internal composition index (fluorescence intensity)	Total fluorescence of intracellular components (e.g., NADH and flavins).	Reflects metabolic activity and cellular health.	Fluorescence spectroscopy.
Lifespan	Average lifespan of erythrocytes (typically 120 days in humans).	Shortened lifespan can indicate premature cell destruction, as seen in hemolytic anemia.	In vivo labeling, tracking biomarkers.

**TABLE III.** Spectral properties of erythrocytes by age group.

Age group	MCH range (pg)
6 months–8 years	24–30
9–11 years	26–32
12–17 years	26–34
18–44 years	27–34

These values give insights into typical excitation and emission wavelengths for erythrocytes and highlight the variety in fluorescence intensity based on the specific conditions and wavelengths used in different studies.

These characteristics enable a comprehensive evaluation of erythrocyte health and functionality, allowing for the identification of pathological conditions based on deviations from typical ranges. Each metric can be measured with specific techniques suited for detailed analysis in laboratory and clinical settings.

This value may vary depending on the individual, age, and health status. The MCH value can be measured separately or obtained from the literature to be used to determine the RBC count.

An age-based chart showing typical MCH (mean corpuscular hemoglobin) levels is shown in Table III.

This table shows how MCH levels increase with age. Children and young adults often have slightly lower MCH values than adults.

Table IV shows summary of the expected MCH variations given the various factors that may affect the MCH value:

**TABLE IV.** Spectral properties of erythrocytes by health condition.

Factor	Impact on MCH	Explanation
Sex	Minor differences	Slightly lower in women due to menstrual blood loss, although generally within the same range as men.
Iron levels	Decreases with deficiency	Iron-deficiency anemia typically leads to low MCH, as iron is essential for hemoglobin synthesis.
Vitamin B12 levels	Increases with deficiency	Vitamin B12 or folate deficiency can cause macrocytic anemia, leading to a higher MCH due to larger RBCs.
Chronic diseases	Variable	Chronic conditions (like kidney disease) can disrupt RBC production, potentially lowering MCH.
Genetic disorders	Variable (often decreased)	Conditions like thalassemia or sickle cell disease affect hemoglobin production, often lowering MCH.
Bone marrow health	Decreased if compromised	Disorders like aplastic anemia reduce RBC production, potentially leading to low MCH.
Alcohol consumption	May increase	Chronic alcohol use can lead to macrocytosis (larger RBCs), raising MCH.
Altitude	Minor increase	High altitudes stimulate more hemoglobin production, potentially increasing MCH slightly.

To estimate the total hemoglobin concentration in blood using a spectrometer, we typically measure the absorbance of blood at a specific wavelength, such as **540 nm**, which is strongly absorbed by hemoglobin. The absorbance value can then be used with the Beer–Lambert law to calculate the concentration. The concentration in mol/L is converted to g/L using hemoglobin's molar mass (approximately 64 500 g/mol).

## V. SPECTRAL AND SIMULATION ANALYSIS OF ERYTHROCYTE PROPERTIES

In this section, we present a series of graphical analyses to illustrate the fluorescence and spectroscopic properties of erythrocytes under various conditions. These graphs visually represent the relationships between key erythrocyte biomarkers, the deformability index, and environmental factors such as oxygen concentration, temperature, osmotic pressure, and electrical characteristics (zeta potential). By examining these visual data, we can observe trends and patterns that contribute to a better understanding of erythrocyte functionality and health status.

**FIG. 2.**
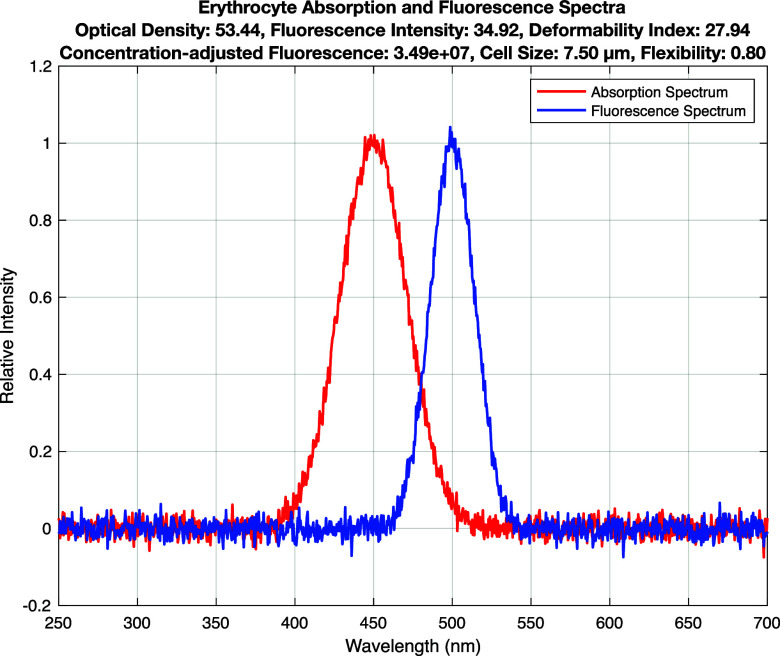
Erythrocyte absorption and fluorescence spectra.

Figure 2 shows the absorption and fluorescence spectra of erythrocytes (red cells). Absorption and fluorescence are concentrated in a defined range of wavelengths. These characteristics indicate the structural and functional state of erythrocytes. A strong fluorescence signal may indicate healthy erythrocytes, whereas a low flexibility index indicates a slower moving and less flexible state of the cells.

**FIG. 3.**
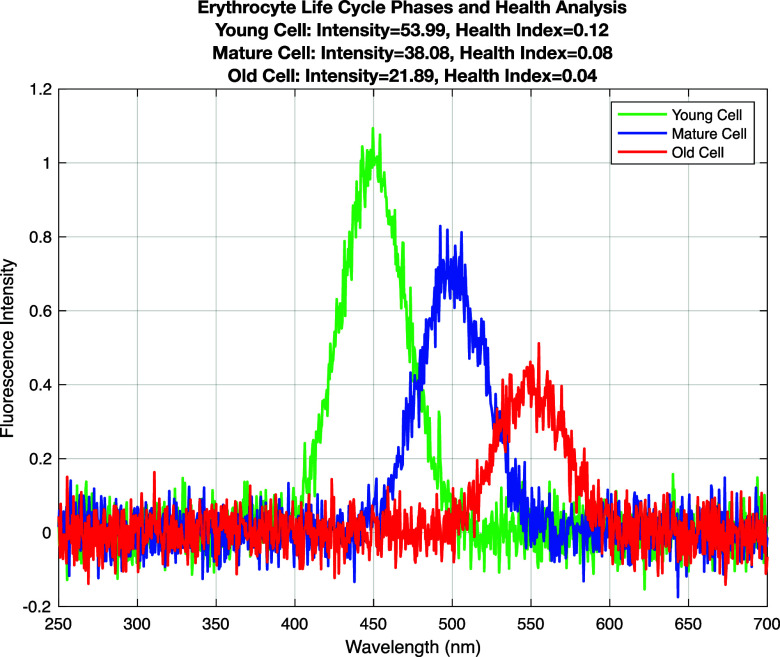
Erythrocyte life cycle phases and health analysis.

Figure 3 describes the different phases of the erythrocyte life cycle: the fluorescence intensity of young, mature, and aged cells. Each phase is shown in a different color: young (green), mature (blue), and old (red). Young cells show the highest fluorescence intensity and a high healthy index level, which indicates their activity and stable state. The intensity of mature cells is decreased, and the fluorescence intensity and health index of aged cells are particularly low, reflecting the senescence and degradation process of cells.

**FIG. 4.**
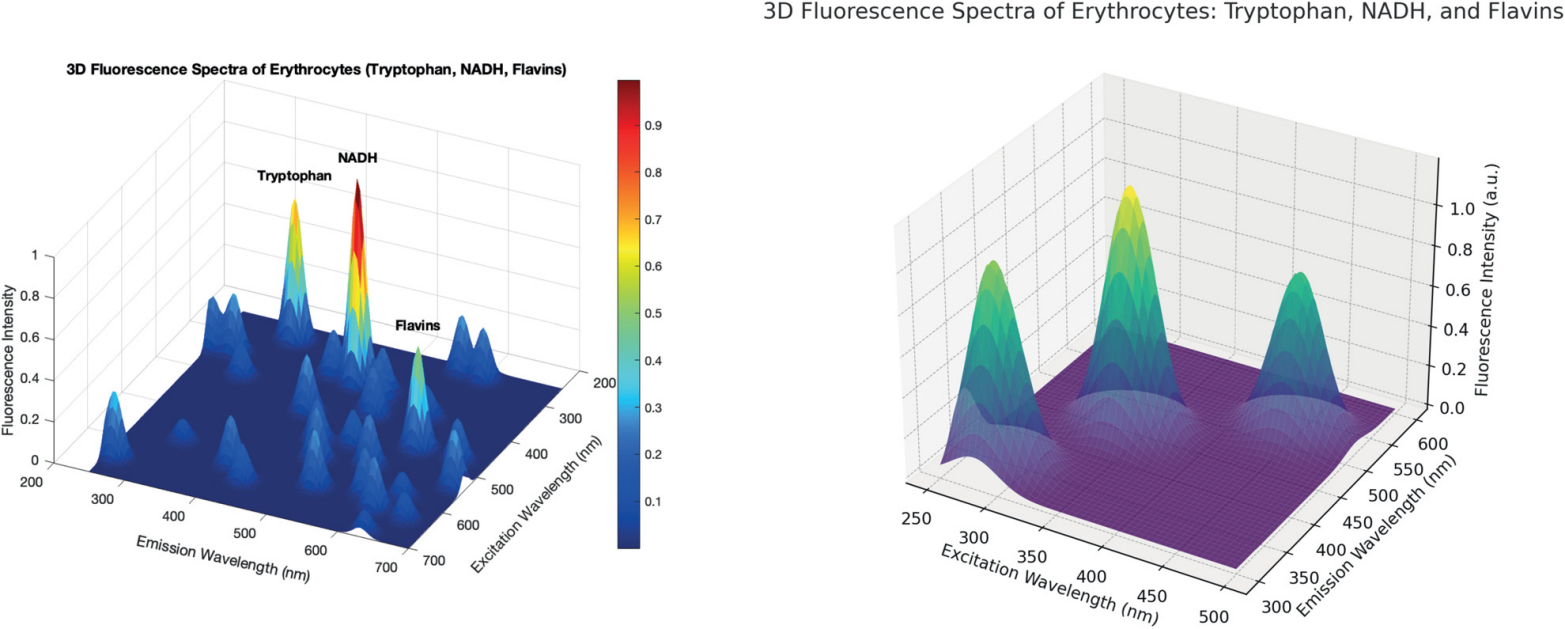
3D fluorescence spectra of erythrocytes (tryptophan, NADH, and flavins).

Figure 4 shows the 3D fluorescence spectra of the main compounds of erythrocytes: tryptophan, NADH, and flavins. The vertical axis represents the fluorescence intensity, and the abscissa and ordinate axes represent the excitation and emission wavelengths. NADH has the highest fluorescence activity and has a central role in the process of energy metabolism. Fluorescence of tryptophan and flavins is at an average level, indicating that they are less active. Overall, the fluorescence profile of these components shows the level of metabolic activity of erythrocytes. Tryptophan often shows a peak around 280–300 nm excitation, with an emission peak around 340–350 nm. NADH has a characteristic excitation peak around 340–360 nm, with emission typically around 450–460 nm. Flavins exhibits peaks at 450–470 nm excitation, with emission around 520–530 nm.

**FIG. 5.**
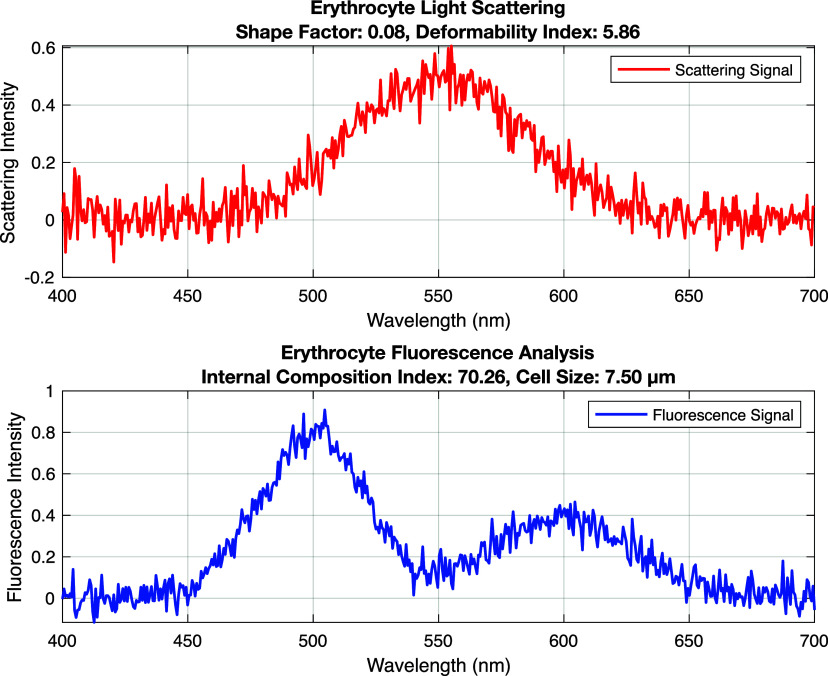
Erythrocyte light scattering and fluorescence analysis.

Figure 5 consists of two parts: the upper graph shows the light scattering (in red), and the lower one shows the fluorescence analysis data (in blue). Characteristics include form factor, deformability index, internal composition index, and cell size. The light scattering peak indicates the stability of the cell shape and its ability to facilitate light distribution. The fluorescence signal indicates the index of the internal composition of erythrocytes and the cell size. Based on these indicators, it is possible to assess the health of the cell and analyze its structure.

**FIG. 6.**
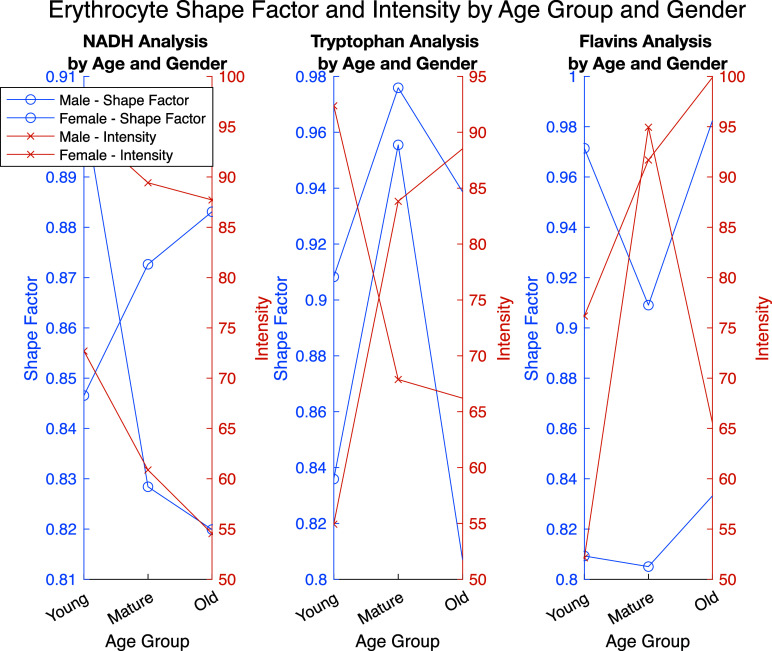
Age and gender analysis of erythrocyte shape factor and fluorescence intensity across key biomarkers.

Figure 6 depicts the erythrocyte shape factor and fluorescence intensity for three key biomarkers—**NADH**, **tryptophan**, and **flavins**—across different age groups and genders. The left y-axis represents the **shape factor**, indicating the structural condition of the cells, while the right y-axis shows **fluorescence intensity**, which reflects metabolic activity. NADH fluorescence intensity varies across age groups, especially by gender. In young cells, both the shape factor and intensity are high, which may indicate increased metabolic activity. Tryptophan intensity decreases with age in both genders. Young cells exhibit a higher tryptophan intensity and shape factor, serving as an indicator of metabolic activity. Flavin intensity also decreases with age, with a notable difference in shape factor variation by gender. In females, flavin intensity declines more significantly with age, suggesting age-related structural changes in cells.

**FIG. 7.**
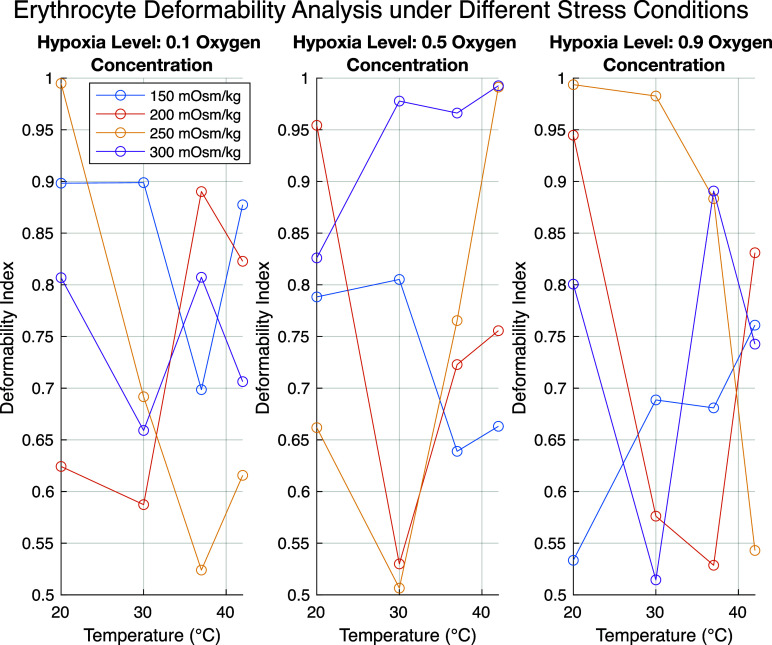
Erythrocyte deformability analysis under varying temperature, osmotic pressure, and hypoxia conditions.

Figure 7 shows the deformability index of erythrocytes under different stress conditions: temperature (°C), osmotic pressure (mOsm/kg), and hypoxia level (oxygen concentration). Each subplot represents a different level of hypoxia, with oxygen concentration fractions of 0.1, 0.5, and 0.9. The y-axis displays the deformability index, which indicates the flexibility of erythrocytes under various conditions, while the x-axis represents temperature. Each line corresponds to a different osmotic pressure. The changes in deformability index (DI) at various levels of hypoxia are evident. At low oxygen concentration (0.1), the DI fluctuates more significantly, which may indicate structural adaptation of cells in response to oxygen deficiency. As temperature increases, the DI generally rises or falls, showing that temperature significantly affects erythrocyte flexibility. At higher temperatures (e.g., 42 °C), the DI decreases under certain pressures, suggesting a potential for structural damage to cells. Different osmotic pressures have distinct effects on deformability. For example, under 150 mOsm/kg pressure, the DI is higher, which may indicate a lower cell compactness.

**FIG. 8.**
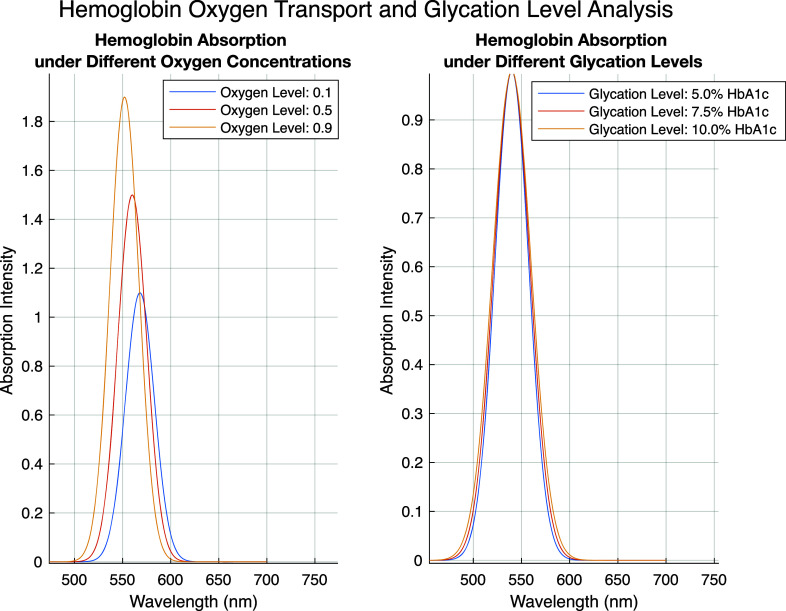
Hemoglobin absorption under different oxygen concentrations (left graph) and under different glycation levels (right graph).

The left graph of Fig. 8 shows hemoglobin's absorption spectrum at different oxygen concentrations (0.1, 0.5, and 0.9). The y-axis represents absorption intensity, and the x-axis indicates wavelength (nm). The right graph shows hemoglobin's absorption spectrum at various glycation levels (% HbA1c)—5%, 7.5%, and 10%. The absorption intensity peaks at approximately 550 nm and increases with higher oxygen concentrations. This suggests that a higher oxygen level results in increased hemoglobin absorption intensity, reflecting enhanced hemoglobin activity for oxygen transport. Thus, this graph provides important insight into the efficiency of oxygen transport. As glycation levels increase (especially at 10%), the absorption intensity decreases and becomes less distinct. This may indicate that glycated hemoglobin is less effective in functioning as normal hemoglobin for oxygen transport. High glycation levels are associated with diabetes, leading to functional limitations in hemoglobin.

**FIG. 9.**
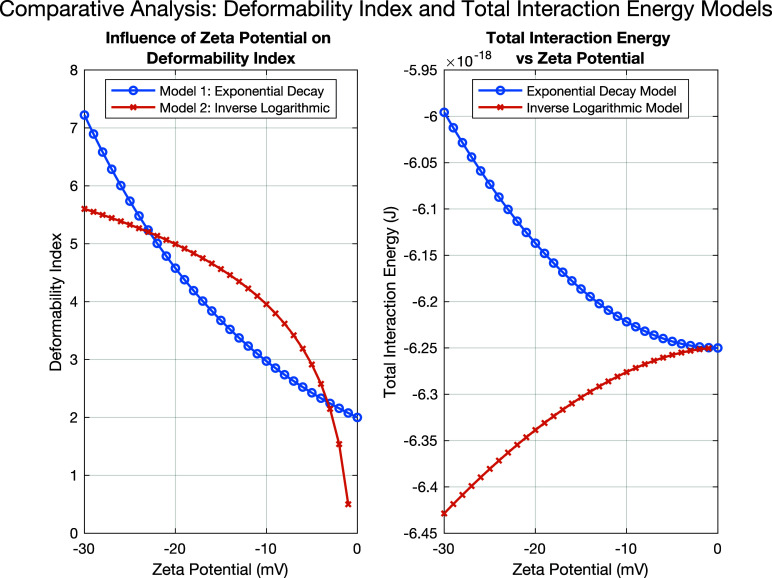
Influence of zeta potential on deformability index (left graph) and total interaction energy (right graph) exponential decay (blue) and inverse logarithmic (red).

Figure 9 displays the influence of zeta potential on deformability index using two models: exponential decay and logarithmic. Let us consider a=1.5—scaling constant for DI responsiveness, b=−0.05—sensitivity to zeta potential, and c=0.5—baseline deformability. The exponential model shows a more gradual and consistent decline, implying a steady decrease in deformability as zeta potential changes. The inverse logarithmic model suggests a sharper decline in deformability once zeta potential is close to zero, potentially indicating that erythrocytes maintain some flexibility up to a certain threshold of zeta potential, after which their deformability sharply drops. This analysis suggests that zeta potential plays a crucial role in maintaining erythrocyte deformability. A highly negative zeta potential (high electrostatic repulsion) supports better deformability, which is essential for erythrocytes to move through capillaries and supply oxygen efficiently. However, as the zeta potential becomes less negative, the cells become more rigid, potentially leading to impaired blood flow and health issues. The models may be useful for predicting deformability changes under various physiological conditions, such as inflammation or certain diseases that affect cell surface charge.

## VI. COMPARATIVE ANALYSIS OF FLUORESCENCE SPECTROSCOPY AND HEMATOLOGICAL PARAMETERS IN TWO PATIENTS

In this section, we present a comparative analysis of the clinical and spectroscopic blood test results for two patients. To ensure a thorough and standardized examination, venous blood samples were collected from both patients under identical conditions. These samples were subsequently diluted using an isotonic phosphate-buffered saline (PBS) solution to maintain their physiological properties while minimizing potential artifacts in the spectroscopic measurements. This dilution process is crucial as it prevents unwanted hemolysis, which could otherwise distort fluorescence signals and influence the accuracy of erythrocyte characterization.

**FIG. 10.**
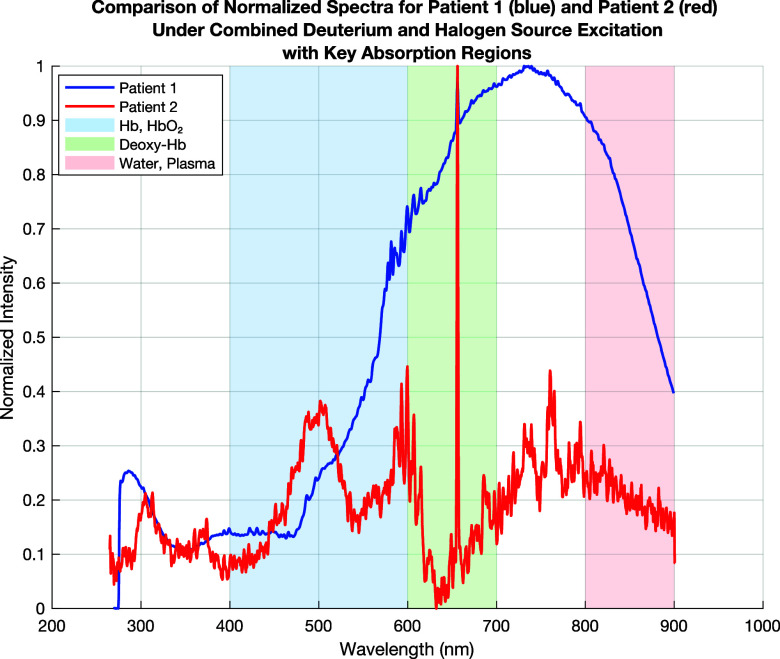
Comparison of spectra for patient 1 (blue) and patient 2 (red) under combined deuterium and halogen source excitation. Hemoglobin and oxyhemoglobin absorption (400–600 nm, blue region), deoxyhemoglobin absorption (600–700 nm, green region), and plasma and water absorption (800–900 nm, red region).

**FIG. 11.**
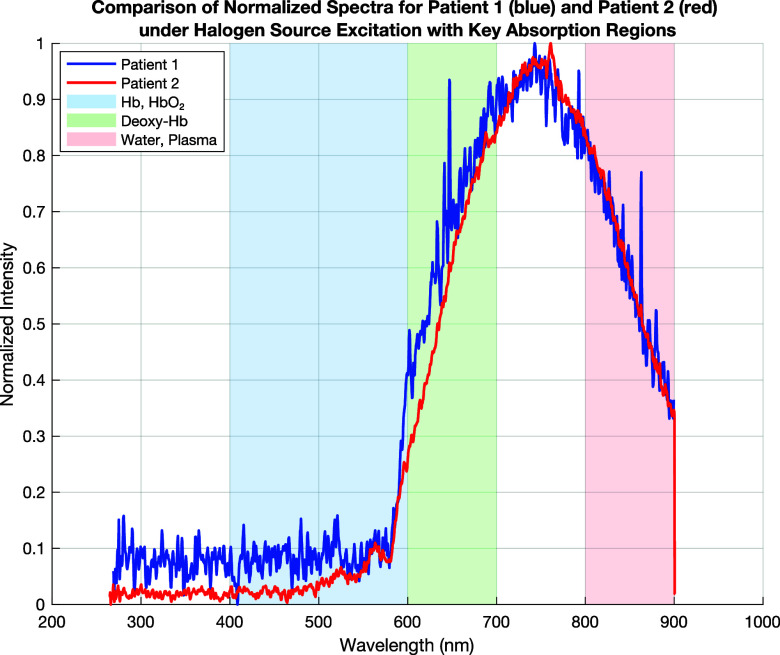
Comparison of spectra for patient 1 (blue) and patient 2 (red) under halogen source excitation. Hemoglobin and oxyhemoglobin absorption (400–600 nm, blue region), deoxyhemoglobin absorption (600–700 nm, green region), and plasma and water absorption (800–900 nm, red region).

**FIG. 12.**
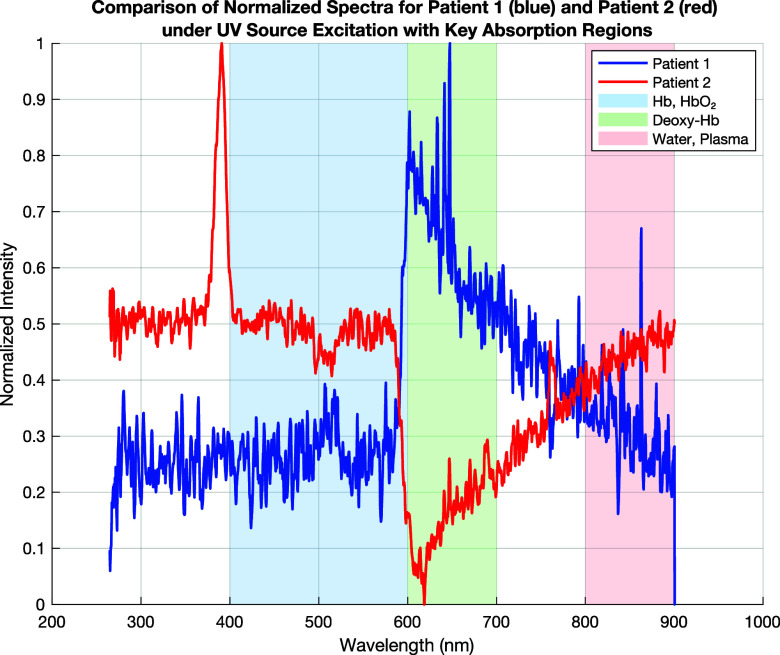
Comparison of spectra for patient 1 (blue) and patient 2 (red) under UV source excitation. Hemoglobin and oxyhemoglobin absorption (400–600 nm, blue region), deoxyhemoglobin absorption (600–700 nm, green region), and plasma and water absorption (800–900 nm, red region).

Parallel to the standard clinical hematological analysis, fluorescence spectroscopy was conducted to explore the optical properties of the blood samples. The motivation for integrating spectroscopic evaluation alongside conventional blood tests stems from the need to enhance diagnostic precision. While clinical tests provide quantitative data on red and white blood cell counts, hemoglobin concentration, and hematocrit levels, fluorescence spectroscopy offers additional insight into erythrocyte biochemical composition. Figures 10–12 present the spectroscopic comparison of the blood samples under different excitation sources, including deuterium and halogen combined excitation, halogen-only excitation, and ultraviolet (UV) excitation. Each spectral analysis highlights key absorption regions corresponding to hemoglobin, deoxyhemoglobin, and plasma content. These results will be discussed in conjunction with the clinical hematology findings to elucidate potential physiological and pathological implications. Through this combined analysis, we aim to establish fluorescence spectroscopy as a complementary tool in hematological diagnostics, enhancing the ability to detect and characterize blood disorders with a greater accuracy, oxygenation status, and molecular interactions. By mapping the fluorescence emission across multiple excitation wavelengths, this method enables the identification of deoxygenated and oxygenated hemoglobin, plasma composition, and metabolic variations.

Figure 10 presents the fluorescence/absorption characteristics of blood samples from two patients under combined excitation using deuterium and halogen lamps simultaneously. The focus is on the key absorption regions of hemoglobin and plasma. The blue line corresponds to patient 1, while the red line corresponds to patient 2.

**Hemoglobin and Oxyhemoglobin Absorption (400–600 nm, Blue Region)** shows that for patient 1 a stronger absorption in this region, especially around 540–580 nm, where Hb and HbO_2_ are most active, but patient 2 displays a lower intensity in this region, suggesting a lower hemoglobin or oxygenated hemoglobin concentration. This means that we have a higher absorption in patient 1, which suggests a higher hemoglobin concentration or better oxygenation (SpO_2_)c, and a lower absorption in patient 2 may indicate anemia or reduced oxygen-carrying capacity.

**Deoxyhemoglobin Absorption (600–700 nm, Green Region)** shows that for patient 1 a lower intensity, implying more oxygenated blood, and for patient 2 a higher absorption in this region, suggesting a higher presence of deoxyhemoglobin (Deoxy-Hb). A higher Deoxy-Hb in patient 2 suggests poor oxygen saturation, possibly hypoxia or a blood circulation issue. A lower Deoxy-Hb in patient 1 suggests normal oxygenation levels.

**Plasma and Water Absorption (800–900 nm, Red Region)** shows that for patient 1 we a have higher intensity in this range, suggesting more plasma or water content and for patient 2 a lower intensity, indicating a higher hematocrit (more red blood cells and less plasma). A higher plasma/water absorption in patient 1 may suggest diluted blood, lower hematocrit, or potential dehydration effects. Lower plasma absorption in patient 2 suggests a higher hematocrit, possibly thicker blood, which could increase clotting risks.

Patient 1 shows normal or slightly anemic blood characteristics with a tendency toward a higher plasma content. Patient 2 may have reduced oxygenation, a higher hematocrit, and a higher risk of clotting or circulatory issues. Clinical blood tests (hemoglobin concentration, hematocrit, oxygen saturation, and RBC count) should confirm these spectral findings. If patient 2 has high Deoxy-Hb and hematocrit, they may need oxygen therapy or hydration treatment (Table V).

**TABLE V.** Comparative summary of blood conditions.

Parameter	Patient 1 (blue)	Patient 2 (red)
Hemoglobin and oxygenation (400–600 nm)	Higher Hb/HbO_2_	Lower Hb/HbO_2_ (possible anemia)
Deoxy-hemoglobin (600–700 nm)	Lower deoxy-Hb (better oxygenation)	Higher deoxy-Hb (possible hypoxia)
Plasma/water absorption (800–900 nm)	Higher plasma content (lower hematocrit)	Lower plasma content (higher hematocrit)

This analysis shows how fluorescence spectroscopy can detect differences in the blood composition, providing valuable noninvasive diagnostic insights.

Figure 11 presents the fluorescence/absorption spectra of two patients' blood samples, but this time excited using only a halogen lamp. The blue line represents patient 1, and the red line represents patient 2. Compared to the previous graph (where both deuterium and halogen excitation were used), several key differences are noticeable.

**Increased Intensity in the 700–900 nm Region for Patient 2 (Red Line):** Patient 2 now shows a significantly higher intensity in the 800–900 nm range, much stronger than in the deuterium + halogen excitation case. Patient 1 also exhibits an increase in this range but at a much lower intensity.

The halogen lamp provides a stronger near-infrared (NIR) excitation, which enhances absorption in the plasma and water absorption region. A higher absorption for patient 2 in this region suggests increased plasma or water content, possibly indicating higher hydration levels or a lower hematocrit (more plasma, fewer red blood cells). This contrasts with the previous analysis, where patient 2 had a higher hematocrit; the difference may arise from different excitation properties of halogen vs deuterium light sources.

**Stronger Deoxyhemoglobin Absorption (600–700 nm, Green Region):** Patient 2 still shows a stronger absorption in the deoxyhemoglobin region (600–700 nm). This confirms the previous finding that patient 2 has a higher level of deoxygenated hemoglobin (Deoxy-Hb). Patient 1 has a lower absorption in this range, reinforcing that oxygen saturation (SpO_2_) is higher.

The presence of strong Deoxy-Hb absorption confirms that patient 2 likely has a lower oxygenation, which could indicate hypoxia, anemia, or circulatory inefficiency.

**Hemoglobin and Oxyhemoglobin Absorption (400–600 nm, Blue Region):** Patient 1 still exhibits a higher absorption in the 400–600 nm range, suggesting higher levels of oxyhemoglobin (HbO_2_). Patient 2 again has a lower intensity in this range, reinforcing the idea of lower oxygen-carrying capacity or a lower hemoglobin concentration.

Consistent with the previous analysis, patient 1 has better oxygenation and likely a healthier blood profile. Lower absorption in patient 2 in this range continues to suggest possible anemia or reduced oxygen transport efficiency (Table VI).

**TABLE VI.** Comparison with the deuterium + halogen spectrum.

Observation	Deuterium + halogen excitation	Halogen excitation only	Possible explanation
700–900 nm region (water and plasma)	Higher in patient 1	Much higher in patient 2	Halogen lamp excites NIR better, revealing stronger plasma/water presence in patient 2.
600–700 nm region (deoxy-Hb)	Stronger in patient 2	Still stronger in patient 2	Deoxyhemoglobin signature remains consistent, confirming lower oxygenation in patient 2.
400–600 nm region (Hb, HbO_2_)	Higher in patient 1	Still higher in patient 1	Confirms patient 1 has better hemoglobin-based oxygenation.

Figure 12 presents the fluorescence/absorption spectra of two blood samples (patient 1—blue and patient 2—red) under UV excitation, compared to the previous results obtained using halogen and deuterium lamps. The UV light source highlights different spectral characteristics, particularly affecting the protein and nucleic acid absorption regions.

**Significant Negative Absorption in the 600–700 nm Region (Deoxy-Hb):** Patient 2 shows a strong downward shift (negative intensity) in the deoxyhemoglobin region, indicating a higher absorption. Patient 1 has a positive intensity in this region, suggesting lower Deoxy-Hb levels. UV light highlights differences in deoxygenated hemoglobin (Deoxy-Hb) more strongly than halogen light. Patient 2's absorption dip in this range confirms hypoxia or poor oxygen saturation. Patient 1's stable behavior suggests better oxygen transport.

**Strong Fluctuations in the 400–600 nm Region (Hb, HbO_2_):** Patient 2 exhibits a significant spike at ∼400 nm, followed by fluctuations. Patient 1 shows a stable Hb/HbO_2_ profile. UV excitation strongly interacts with nucleic acids and proteins, which may explain the fluctuations. The large peak at ∼400 nm for patient 2 may indicate oxidative stress, protein denaturation, or abnormal hemoglobin behavior.

**More Stable Behavior in the 700–900 nm Region (Water, Plasma):** Under UV excitation, patient 1 maintains a relatively stable profile, while patient 2 shows some fluctuations. Compared to the halogen excitation, where patient 2 had significantly a higher water absorption, here the difference is less pronounced. UV excitation does not enhance the water absorption signature as much as halogen or NIR light. Patient 1 still appears to have a more balanced plasma-to-cell ratio, while patient 2 may have fluctuations due to blood composition changes.

This analysis shows how different light sources reveal unique blood characteristics, improving the accuracy of noninvasive diagnostics (Table VII).

**TABLE VII.** Comparative summary of blood characteristics.

Parameter	Patient 1 (blue, UV excitation)	Patient 2 (red, UV excitation)
Hemoglobin (Hb, HbO_2_) absorption (400–600 nm)	More stable, better oxygen transport.	Strong fluctuations, possible protein, or oxidative effects.
Deoxyhemoglobin (600–700 nm)	Lower absorption, good oxygenation.	Strong absorption dip, possible hypoxia.
Plasma/water absorption (700–900 nm)	More stable, balanced hydration.	Some fluctuations, less prominent than halogen case.

When comparing the clinical blood test (CBC) results with the fluorescence spectroscopy data, we observe both correlations and discrepancies. Table VIII shows an analysis of where these two diagnostic approaches align and where they show notable differences.

**TABLE VIII.** Key differences between CBC and spectral data.

Aspect	Findings in CBC (hematology data)	Findings in fluorescence spectroscopy	Possible causes of discrepancy
Oxygen transport (hemoglobin and RBCs)	-Patient 1 has a higher hemoglobin (16.4 g/dL), a higher RBC count (5.32 × 10^6^/*μ*L), and a higher hematocrit (46.9%), suggesting good oxygen transport. -Patient 2 has a lower hemoglobin (12.6 g/dL), a lower RBC count (4.36 × 10^6^/*μ*L), and a lower hematocrit (38.5%), suggesting mild anemia.	-Despite lower hemoglobin, patient 2 shows a strong spectral signal at 600–700 nm (deoxyhemoglobin range). —Patient 1 has a stronger oxyhemoglobin fluorescence signal at 540–580 nm, which aligns with their CBC data.	-Patient 2's strong deoxyhemoglobin fluorescence may indicate localized hypoxia or altered oxygen dissociation rather than a systemic RBC deficiency.
Plasma vs. RBC ratio (hydration and hematocrit influence)	-Patient 1 has a higher hematocrit, meaning a higher RBC concentration and a lower plasma volume. -Patient 2 has a lower hematocrit, indicating a relatively higher plasma-to-RBC ratio.	-Stronger plasma/water absorption in the 700–900 nm range is seen in patient 2. -Patient 1 shows less water absorption, correlating with a higher RBC ratio.	-Fluorescence spectroscopy captures plasma composition more prominently than CBC, emphasizing hydration levels and plasma-bound biomolecules.
Inflammation and immune response	-Patient 2 has significantly higher neutrophils (65.4%), suggesting acute inflammation or bacterial infection.-Patient 1 has more lymphocytes (37.3%), suggesting a possible viral infection or chronic immune response.	-No direct correlation between immune markers and fluorescence spectra. -However, spectral variations in the UV range (proteins, oxidative stress markers) may relate to immune activation.	-Spectroscopy does not directly measure WBCs, but oxidative stress and metabolic byproducts may influence UV spectra.
Metabolic and oxidative stress indicators	-Patient 1 has increased eosinophils (7.2%) and basophils (4.2%), potentially indicating an allergic or inflammatory response. - Patient 2 does not show this pattern.	-UV spectra of patient 1 show possible signs of protein oxidation, which may align with immune activation. –Patient 2 has a lower fluorescence in these regions.	-Oxidative stress biomarkers are detected in fluorescence but are not explicitly measured in CBC.
Anemia and oxygen dissociation	-Patient 2's lower hemoglobin and RBC count indicate mild anemia.	-However, the fluorescence signature of hemoglobin suggests higher deoxyhemoglobin signals, meaning oxygen delivery issues rather than pure RBC deficiency.	-CBC indicates total RBC and hemoglobin levels, while fluorescence spectroscopy reveals real-time oxygen dissociation and binding efficiency.

CBC provides **quantitative blood composition data**, while fluorescence spectroscopy reveals **functional and metabolic insights**, particularly in **oxygen binding, oxidative stress, and plasma composition**. Together, they offer a **comprehensive diagnostic picture that neither method can fully achieve alone**.

## VII. CONCLUSION

We can get information about the morphology of erythrocytes based on scattering intensity and cell diameter (shape factor), cell flexibility (deformability index), and cell geometrical characteristics (size, internal composition index)

3D fluorescence spectra allow us to: observe erythrocyte fluorescence characteristics across different wavelengths, spatially understand the precise micro-/nanostructure and composition of erythrocytes, and identify membrane and structural changes related to health and various pathological processes.

Overall, fluorescence intensity and light scattering data provide a basis for evaluating different states of cells (young, mature, old), as well as for studying their internal metabolism and structure. Based on this, a more in-depth analysis of the condition of erythrocytes is possible, which provides us with additional information on their efficiency and life cycle.

Based on the findings of this study, potential future directions include the exploration of additional biomarkers, which would expand the diagnostic capabilities of fluorescence spectroscopy. Special attention could be given to biomarkers that more accurately reflect pathological conditions or age-related and physiological changes in the organism.

Furthermore, the methodology used in this study could be applied to the analysis of other cell types, opening the door to a broader range of diagnostic research. Analyzing different types of cells would offer new perspectives for fluorescence spectroscopy and significantly broaden its potential in medical diagnostics.

The integration of these approaches aligns with the broader objectives of this study, which emphasize the potential of fluorescence spectroscopy as a noninvasive diagnostic tool. Traditional hematological assessments, while highly informative, may not always capture dynamic physiological variations in oxygen transport, hydration levels, and oxidative stress. In contrast, fluorescence-based methods provide a real-time functional perspective, detecting subtle differences in hemoglobin states, erythrocyte deformability, and plasma composition that may not be apparent through standard hematological parameters.

## VIII. METHODS

### A. Theoretical and simulation modeling

This study is based on established theoretical models of erythrocyte microrheology, fluorescence spectroscopy, and light absorption/emission principles in biological systems. The primary theoretical frameworks incorporated into this research include the following:

• The study relies on the principles of Jablonski diagrams, fluorescence lifetime, and emission-excitation matrices to analyze erythrocyte fluorescence signals.
• Hemodynamic properties and deformability indices were assessed using models describing zeta potential influence on erythrocyte aggregation and deformability.
• The analysis of hemoglobin absorption spectra was based on the Beer–Lambert law and Mie scattering principles.
• Principal component analysis (PCA) and Gaussian fitting were applied to classify spectral data and identify fluorescence contributions from key biomolecules such as tryptophan, NADH, and flavins.

To validate theoretical predictions, simulation modeling was conducted in MATLAB. The simulations included the following:

• Normalization of fluorescence spectra to standardize intensity variations.
• Comparative analysis of experimental spectra with simulated reference spectra.
• Peak identification and biomarker quantification for hemoglobin derivatives and plasma components.
• Multivariate data analysis to assess the influence of different excitation wavelengths.

These simulations helped refine the experimental setup and optimize data interpretation for fluorescence-based hematological diagnostics.

### B. Sample preparation and collection

Venous blood samples were collected from two patients at Batumi Shota Rustaveli State University's affiliated laboratory under standard clinical conditions. The samples were diluted in phosphate-buffered saline (PBS) to maintain physiological conditions and prevent hemolysis, ensuring reliable spectroscopic measurements.

### C. Fluorescence spectroscopy setup

Fluorescence spectroscopy was performed using a BlackComet spectrometer manufactured by StellarNet (www.stellarnet.us). The excitation sources included deuterium, halogen, deuterium + halogen, and UV lamps. Spectral data were collected within a 200–900 nm range, hemoglobin and oxyhemoglobin absorption (400–600 nm, blue region), deoxyhemoglobin absorption (600–700 nm, green region), and plasma and water absorption (800–900 nm, red region) with emission wavelengths recorded at 280 nm (tryptophan), 340 nm (NADH), and 460 nm (flavins).

### D. Data processing and normalization

The collected spectral data were normalized using maximum intensity scaling to compare patient samples effectively. A baseline correction algorithm was applied to remove the background noise, and data were smoothed using a Gaussian filter. The initial spectra were recorded using the SpectraWiz software, while the normalization and comparison of the spectra were performed in MATLAB.

### E. Clinical blood analysis

Routine hematological parameters, including hemoglobin concentration, hematocrit, and red blood cell count (RBC count), were measured using an automated blood analyzer. These values were then correlated with the fluorescence spectroscopy results to determine any diagnostic significance.

## Data Availability

The data that support the findings of this study are available from the corresponding author upon reasonable request.
